# A Weak Spot in the Popular Religion

**Published:** 1891-11-28

**Authors:** 


// The Hospital

A Weekly Journal of

Science, Medicine, Nursing, and Philanthropy.

*For Hospitals, Asylums, Infirmaries, Dispensaries, Medical Practitioners, Students, Nurses, and the Charitable Public.*

Vol. XI.—No. 270.] [Nov. 28, 1891.

### The Hospital

### A Weak Spot in the Popular Religion.

Is the Christian Church a failure? This is not the same as asking if Christianity is a failure. Our object in discussing the subject is not critical, but practical. In order to answer the question, Is the Christian Church a failure? it is necessary to consider what is the chief purpose of the Church's existence and activity. It is desirable to point out that an institution like the Church may have more than one object, may, indeed, have several; and that two or more of them may be of almost equal importance. This, in fact, we find to be the case with the Christian Church. It has several great purposes to fulfil; and it is quite possible to conceive that whilst it may be entirely successful in some, it may fail partially or wholly in others.

There are few who will deny that it is one of the most important functions of the Church to supply a concrete organisation and a ritual which shall meet and satisfy the natural religious aspirations of the people. Man has been defined as a worshipping animal. The definition is hard, unpoetical, mechanical, and lean. It is literally correct in a limited sort of way, but it gives a miserable account of the facts of the case. Man is made with an insatiable desire for knowledge. He explores wherever he can explore, and where he cannot explore he speculates and guesses. But curiosity is almost the weakest of all his religious motives. The average man lives but a very short time in the world before he discovers in himself a mixed nature. Part of him aspires after intellectual and moral justice; is conscious of sympathy, not only with eternal order, but with what we call purity, moral beauty, and a magnanimity altogether beyond what can be found in the average or even in the exceptional man But men, and women still more, very early in their experience become conscious also of personal defeats, disappointments, and failures; the soul experiences strokes of adversity, and is bruised by them: it feels hurt and wounded.

There is thus created a necessity for the utterance of distress, of disappointment; a longing for sympathy, accompanied often by an enkindling of hope. Some individual souls can express all these things at a shrine of devotion, all alone and without companionship. But others cannot do this; they need to be led, and to feel themselves in company. The priest is indispensable to them, and the great congregation, and the sacraments. We are not here considering the scientific value of religion, or the possibility that at some remote future in the world's history it may be altogether dispensed with. We are merely taking the facts as we find them, and as the world has always found them within historic memory.

The Christian Church, if it were to continue to exist at all, was bound to furnish an organisation which should gather to a focus, and give manifold voice to the natural religious aspirations of the people. This the Christian Church has done, and done admirably; more especially, we think, during the last three centuries. It has furnished venerable temples for worship, simple and devout forms, a beautiful and expressive liturgy, solemn and affecting rites for childhood, for marriage, and for departing spirits as they pass into the great unknown. Was it not well that these things should be done? Was it not indispensable? How could we have had an ordered civilisation if external religion had been a chaos and an anarchy? Science—the science of man, at any rate—must think of all these things. Can it be questioned for a moment that in supplying to us a sympathetic ritual, and a beautiful pathway towards the infinite, the Christian Church has succeeded, not only better than any other religious organisation, but almost beyond all expectation and hope?

In a scientific age the Church is, above all things, under the necessity of being reasonable. This is no light demand to make upon an organisation in which faith plays, and must play, so markedly conspicuous a part. Although few living men have seen the phenomenon of faith removing mountains, yet how filled we all are, who have any faith at all, with the consciousness that it is the nature of faith to remove mountains; and that the mechanical laws which govern our present existence are prison bars which faith is constantly endeavouring to break through, and which some day it will burst asunder and gloriously triumph over, as if those laws had never been in operation at all. But, notwithstanding these undoubted endowments and strivings of the natural mind of man, a scientific age finds its chief work in quite other regions than those which faith delights to expand itself in. It is imperative in such an age that religion, embodied in the Church, shall speak to the reasonable side of man, and speak so as to carry conviction. If it do not, we are threatened with that direst of all conceivable calamities—universal Atheism.

How has the Church met, and how does she continue to meet, this demand for a reasonable religion? She meets it, happily for the world, in a reasonable and hopeful spirit. A Butler, a Taylor, a Chalmers, a Stanley, a Tait—those and suchlike have been the men who have met science with science, reason with reason, and humanity with humanity. The temper of mind that has animated nearly all the most distinguished religious leaders of the last two centuries has been entirely worthy, at once of the highest science and of the noblest religion. In this also the Christian Church has been conspicuously successful.

But wherein, then, has the Christian Church failed, if she has failed at all? She has failed exactly where it was always probable that she would fail, but where she could least afford to fail. She has failed in practice—alas! for her, that it should be so; and alas! for human nature that such failure was from the first so inevitable! It is but the few who take delight in a beautiful ritual and simple worship; it is a still smaller number who respond to the sober claims of a reasonable religious theory of the universe. The thing that appeals to a gross, hurrying, and intellectually childish world is concrete benevolence—intelligent good-will in active and daily operation. That is where the Christian Church, even in our England, is so conspicuously, we had almost said, disgracefully at fault. Nearly every daily newspaper is a candle which shines upon the outward form and daily doings of the organisation which calls itself the Christian Church. But last week poor Haynes, the cooper, died of starvation after vainly endeavouring to find work. Since his death a woman at Deptford, with three shillings a-week from the parish, and three children and herself to support by it, was tempted to pawn a shirt which had been entrusted to her for washing. She pawned the shirt, but could not redeem it when the time came. This was the last stroke of fate that drove her to despair. She put her "marriage lines" into her pocket to show that she had lived a virtuous woman, and then drowned herself. A man at Stepney, named Gold, was so poor that he also died of starvation. A poor woman expired in a fit at the end of the week. She had sometimes had as many as eight fits a day. This had been her state for years, but not once had she ventured upon the effort even to obtain medical relief. Three of these stories appeared in one paragraph of a daily newspaper last Monday. They are samples of what is occurring every day in this country which is all but exclusively Christian. The Church as a Church, and as, including all Christian denominations, co-extensive with the State, has failed here. It need not be denied—it is painfully, undeniably, terribly true. Why has she failed? Does England fail in business? Does she fail in arms? She fails in neither. Why, then, does she fail in these most elementary articles of her faith? Does she fail because she is Christian? If she does, then let her give up her Christianity, and take to another faith, or to no faith at all. Or does she fail because she is something of a hypocrite, and only professes Christianity, without being at the trouble to understand or practise it? This last is the sad and sober reason. Christianity is not at fault, but only we English men and women ourselves. Is it not time that we took this subject to heart in an honest and scientific way, and learnt the practice of our religion, as well as the preaching and the praying of it? Whatever other religions may be, Christianity is essentially practical, and for daily use. The great call to its professors is not to save their own souls, but to give themselves away, as far as is soberly possible, for the immediate and permanent advantage of their neighbours. It is a high calling, but as honourable as it is difficult and high. Is the English nation capable of rising to so noble a distinction?

